# Contribution of advanced neuroimaging in diagnosis of cerebral syphilitic gumma: a case report

**DOI:** 10.3389/fnins.2024.1442176

**Published:** 2024-08-14

**Authors:** Xinyi Shen, Zhengyang Zhu, Xin Li, Wen Zhang, Xin Zhang, Bing Zhang

**Affiliations:** ^1^Department of Radiology, Affiliated Hospital of Medical School, Nanjing Drum Tower Hospital, Nanjing University, Nanjing, China; ^2^Institute of Medical Imaging and Artificial Intelligence, Nanjing University, Nanjing, China; ^3^Medical Imaging Center, Affiliated Drum Tower Hospital, Medical School of Nanjing University, Nanjing, China; ^4^Jiangsu Key Laboratory of Molecular Medicine, Nanjing, China; ^5^Institute of Brain Science, Nanjing University, Nanjing, China

**Keywords:** cerebral syphilitic gumma, magnetic resonance imaging, Advanced neuroimaging, intracranial neoplasm, case report

## Abstract

**Background:**

Cerebral syphilitic gumma is a rare intracranial infectious disorder. Without a clear history of syphilis and comprehensive serological examinations, it’s challenging to diagnose it accurately prior to surgery through routine magnetic resonance imaging (MRI). Advanced neuroimaging techniques have been widely used in diagnosing brain tumors, yet there’s limited report on their application in cerebral syphilitic gumma. This report presents a case of an elderly male patient with cerebral syphilitic gumma and analyzes its characteristics of advanced neuroimaging.

**Case presentation:**

A 68-year-old male patient was admitted to our institution presenting with bilateral hearing loss complicated with continuing headaches without obvious cause. Laboratory tests indicated positive treponema pallidum. Conventional MRI showed nodules closely related to the adjacent meninges in bilateral temporal lobes. The patient underwent surgical resection of the nodule in the right temporal lobe due to the mass effect and the final pathological diagnosis revealed cerebral syphilitic gumma.

**Conclusions:**

With the return of syphilis in recent years, accurate diagnosis of cerebral syphilitic gumma is a matter of great urgency. Advanced neuro-MRI can serve as a significant complement to conventional MRI examination.

## Introduction

Neurosyphilis is caused by the invasion of the central nervous system by Treponema pallidum, affecting various parts such as the brain, spinal cord, meninges and blood vessels. It can occur at any stage of syphilis infection (He et al., 2021). It can be classified into five subtypes based on clinical symptoms: asymptomatic neurosyphilis, meningeal syphilis, meningovascular syphilis, parenchymatous neurosyphilis (general paresis and tabes dorsalis), and cerebral syphilitic gumma (Chow, 2021). Cerebral syphilitic gumma is relatively rare, characterized by granulomatous formation formed by localized inflammatory reactions in the meningeal artery after syphilitic invasion of the central nervous system. The clinical symptoms are diverse and nonspecific, while the imaging manifestations are atypical, potentially leading to misdiagnosis or missed diagnosis. In recent years, the widespread application of advanced neuroimaging techniques in the diagnosis and differential diagnosis of brain leisions has provided new perspectives for distinguishing cerebral syphilitic gumma from other conditions. This study reports a case of cerebral syphilitic gumma with comprehensive neuroimaging examinations.

## Case presentation

A 68-years-old male patient experienced bilateral hearing loss accompanied by continuous headache without apparent cause for 2 months. The discomfort could be alleviated by rest, with no apparent dizziness, nausea, vomiting, limb weakness, or seizures. He engaged in several high-risk unprotected sexual behaviors over past ten years. MRI performed at the local hospital was suggested granulomatous lesions. Neurological physical examination revealed normal finding. The serological test results revealed positive treponema pallidum antibody with a positive rapid plasma regain (RPR) titer of 1:128.

**FIGURE 1:**
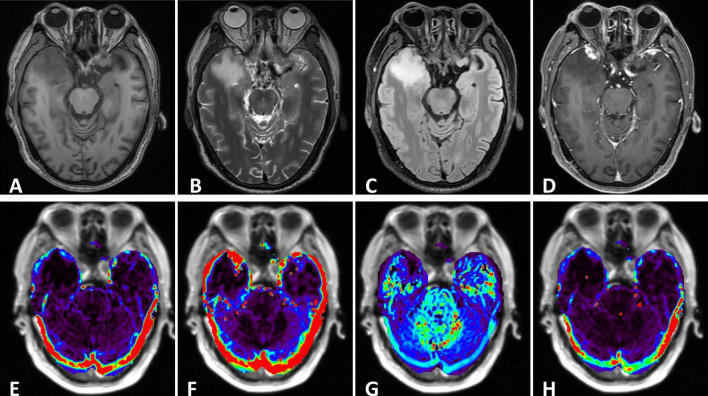
**(A–C)** The right temporal lobe showed a nodular which was characterized by hypointensity on T1 and hyperintensity on T2, with significant edema zone. There also showed slightly edema without clear lesion in the left temporal lobe. The cysts didn’t suppress on FLAIR. **(D)** Enhanced axial T1-weighted image showed significant enhancement of lesions in the bilateral temporal lobes. The adjacent meninges were enhanced, with a characteristic “dural tail”. **(E–H)** DCE parameters analyzed based on the Tofts model: Ktrans, Kep, Ve and iAUC.

**FIGURE 2:**
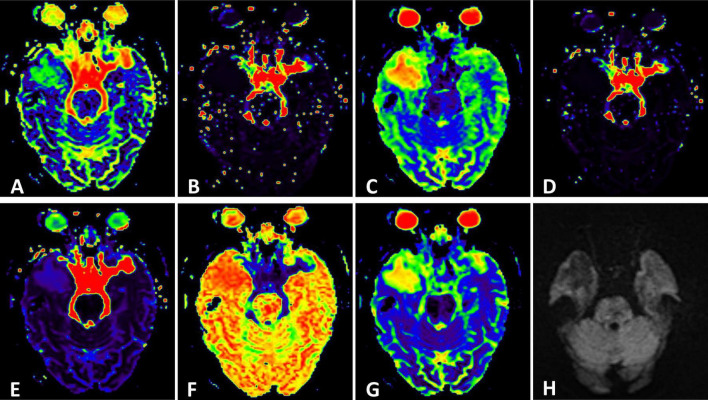
**(H)** Conventional Diffusion-Weighted Imaging (DWI) (b = 1000) shows the lesion with iso-signal. **(A)** F map, **(B)** D map, **(C)** D map, **(D)** rBF map from intravoxel incoherent motion (IVIM) image. **(E)** DDC map, **(F)** α map, **(G)** sADC map from stretched exponential model (SEM) image.

Conventional MRI Perfusion-weighted MRI, dynamic contrast enhanced MRI (DCE-MRI), MR spectroscopy, chemical exchange saturation transfer (CEST) and multiple-b value diffusion weighted imaging sequences were obtained (Supplementary Data Sheet 1). The routine MRI (Figures 1A–D, 2) revealed a nodule (15 × 14 × 12mm) in the right temporal lobe accompanied by surrounding brain tissue edema, presenting as hypointensity on T1WI, hyperintensity on T2WI, and no restricted diffusion on diffusion-weighted imaging. A small patchy edema band was noticed in the left temporal lobe, but the lesion was indistinct. The midline remained was centered, and ventricle system were pressed. Conventional enhanced MRI displayed significant enhancement in the right temporal lobe lesion with enhancement in the adjacent meninges, forming the ‘meningeal tail sign’. A notably enhanced nodule (5 × 4 × 2mm) was also observed in the left temporal lobe after enhancement, closely related to the adjacent meninges.

**FIGURE 3:**
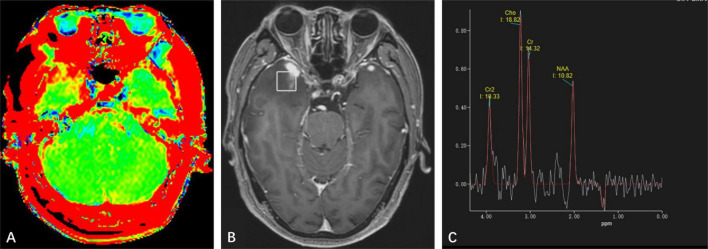
**(A)** Amide proton transfer-weighted imaging shows increased signal on the right side of the lesion, while the signal on the left lesion is consistent with normal brain tissue. **(B)** Positioning image of single-voxel magnetic resonance (MR) spectroscopy. **(C)** Magnetic Resonance Spectroscopy Imaging shows a high Cho peak, a low Cr and NAA peak, Cho/NAA ratio greater than 1, and a negative Lip and Lac peak was observed at 1.33 ppm.

MR spectroscopy (Figure 3) revealed a slight increase choline (Cho) peak and a slight decrease in the peaks of creatine (Cr) and N-acetylaspartate (NAA). The Cho/Cr ratio was 1.31 while Cho/NAA ratio was 1.72. CEST imaging (Figure 3) indicated a higher signal in the larger lesion on the right compared to the signal in normal brain tissue. The smaller lesion on the left exhibits a signal consistent with normal brain tissue. The mean Ktrans, Kep and iAUC in the right lesion were lower than in the contralateral area (Figures 1E–H and Table 1). With SEM and IVIM imaging, the mean parameters of the nodule in the right temporal lobe were higher than those in normal brain tissue, except for the mean f (Figure 2 and Table 2).

**TABLE 1:** Cerebral hemodynamic parameters of the ROI of the lesion and normal control.

Parameter	ROI of the lesion		ROI of normal control	
	Mean	SD	Mean	SD
Ktrans (10^–3^/min)	7.4	6.0	38.7	74.2
Kep (10^–3^/min)	85.6	105.9	756.0	506.6
Ve (10^–3^)	350.1	700.3	43.1	59.0
iAUC (10^–3^min⋅mmol/l)	2.2	1.9	8.5	13.7

Ktrans indicates the volume transfer constant; Kep indicates the time constant of gadolinium reflux; Ve indicates the EES volume per unit tissue volume; iAUC indicates the initial area under the time-concentration curve for the first 60 seconds.

**TABLE 2:** Parameters of IVIM and SEM of the ROI of the lesion and normal control.

Parameter	ROI of the lesion		ROI of normal control	
	Mean	SD	Mean	SD
F (10^–3^)	225.1	95.7	227.2	64.4
D* (10^–5^mm^2^/s)	1107.9	2376.2	722.1	1296.1
D (10^–6^mm^2^/s)	883.5	139.5	546.8	52.1
rBF (10^–5^mm^2^/s)	234.3	562.6	169.5	341.0
DDC (10^–6^mm^2^/s)	1231.4	707.9	761.6	105.3
α (10^–3^)	848.0	106.8	758.8	92.8
sADC (10^–6^mm^2^/s)	951.6	149.0	609.4	48.4

*F* indicates the IVIM-based perfusion fraction; D and D* indicate diffusion coefficient and pseudo-diffusion coefficient; rBF indicates relative blood flow; DDC indicates distributed diffusion coefficient; α indicates water molecular diffusion heterogeneity index; sADC indicates SEM-based apparent diffusion coefficient.

**FIGURE 4:**
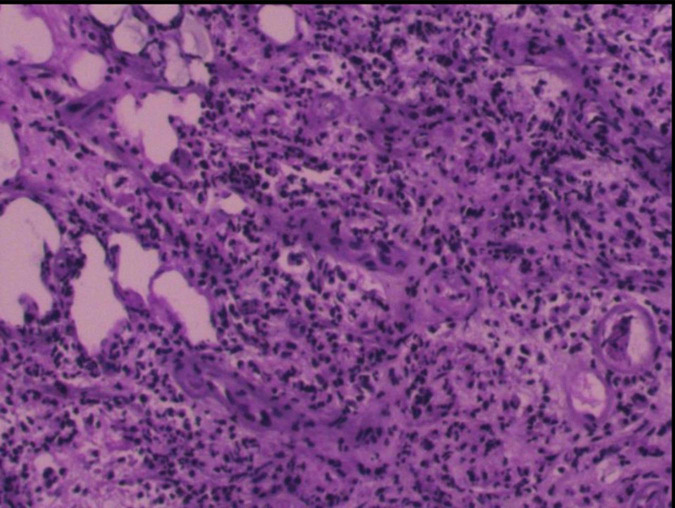
Postoperative pathology reveals fibrous tissue proliferation accompanied by numerous lymphocytes and plasma cells, scattered focally within granulomatous-like structures. Endothelial cell proliferation is observed in small arteries within the lesion, leading to luminal narrowing/solidification. Lymphoplasmacytic infiltration surrounds the vascular walls, histologically consistent with an inflammatory process.

Given the clear history of visiting prostitutes and high titers of treponema pallidum antibody, along with the MRI examinations, cerebral syphilitic gumma was suspected primarily. Considering the size and significant mass effect of the lesion in the right temporal lobe, after excluding contraindications, the patient underwent surgical resection of the nodules. Intraoperatively, brain tissue from the right temporal pole was excised, revealing a lesion located at the temporal base, pale yellow in color, with a clear boundary from the brain tissue and significant edema in the surrounding brain tissue. The postoperative pathological results (Figure 4) revealed granulomatous structures accompanied by fibrous tissue proliferation, surrounded by lymphocytes and plasma cells. This confirmed the diagnosis of cerebral syphilitic gumma.

## Discussion

Syphilis is a chronic infectious disease caused by Treponema pallidum, which can affect multiple organs and systems throughout the body. Syphilis is primarily transmitted through sexual contact. Neurosyphilis is the result of the accumulation of central nervous system involvement. In the early stages of syphilis, Treponema pallidum can penetrate the blood-brain barrier and invade the cerebrospinal fluid or the central nervous system (Chow, 2021). Before the 1950s, neurosyphilis had a high incidence, but with the widespread use of penicillin, neurosyphilis became nearly eradicated. In recent years, syphilis has experienced a resurgence, attributedto factors such as the prevalence of HIV/AIDS, shifts in attitudes toward same-sex and extramarital sexual behavior, and drug use (Chao et al., 2006).

Cerebral syphilitic gumma is extremely rare, typically occurring in the late stage of neurosyphilis, it is classified as a delayed hypersensitivity reaction. in the early stage of syphilis, treponema pallidum primarily involves the meninges or blood vessel walls, resulting in small lesions. If left untreated, obliterative arteriopathy leads to infiltration of inflammatory cells, glial proliferation, and the formation of local granulomas. Central necrosis appears as a caseous coagulative type, surrounded by lymphocytes, plasma cells, polymorphous lymphocytes, and collagen tissue. There is a notable mass effect, and a pronounced edematous zone develops around the lesion (Li et al., 2019). If cerebral syphilitic gumma extensively involves the meninges, it can cause thickening of the meninges, fibrous tissue proliferation, eventually leading to pachymeningitis (Kupersmith et al., 2004).

The symptoms of cerebral syphilitic gumma are nonspecific, mainly determined by its size, location, and compression on adjacent tissues. The complex and variable clinical symptoms, insufficient knowledge of healthcare professionals about this condition, combined with the possibility of patients concealing their medical history during consultation, can lead to misdiagnosis or missed diagnosis of cerebral syphilitic gumma. The diagnosis of neurosyphilis mainly relies on serum and cerebrospinal fluid tests (Li et al., 2019). Establishing the relationship between intracranial lesions and neurosyphilis remains a major challenge in diagnosing cerebral syphilitic gumma. Pathological examination can confirm cerebral syphilitic gumma but requires surgical specimens. To avoid unnecessary surgery, a comprehensive analysis of serological tests, cerebrospinal fluid tests, imaging examinations, and clinical symptoms is often needed.

Conventional MRI examination cannot differentiate cerebral syphilitic gumma from inflammatory granulomas, brain metastases, and malignant meningiomas, but they still present some characteristic features. Lesions can occur in any part of the brain tissue, most frequently on the convex surface of the cerebral meninges, predominantly affecting the gray matter. Routine MRI scans mainly show long T1 and long T2 signals, with a central necrotic area exhibiting low or mixed signals, significant surrounding edema, no diffusion restriction on DWI, and appear as enhancing nodule or circular after enhancement. Some case with uncommon ring enhancement has been reported, necrosis at the nodule center surrounded by epithelial cells, multinucleated giant cells, lymphocytes, and granulation tissue may be a possible explanation (Weng et al., 2019; Takahashi et al., 2022; Mu et al., 2024). Cerebral syphilitic gumma invades the meninges and is closely related to them. The lesion edge often intersects with the surrounding meninges at obtuse angles. There is linear enhancement of adjacent meninges, similar to the “meningeal tail sign,” which is the most significant imaging feature of cerebral syphilitic gumma. Pathological meninges enhancement can also be in other infectious involving the meninges such as bacterial, viral or fungal meningitis, with the pattern of extensive enhancement and meningitis is more likely to present with leptomeningeal enhancement rather than pachymeningeal enhancement. Utoimmune conditions such as sarcoidosis may also produce pachymeningeal enhancement, with about 40% of patients also demonstrating leptomeningeal enhancement. They often spared the convexities of the cerebral hemispheres, which are just the predilection sites of cerebral syphilitic gumma (Antony et al., 2015). The pathological basis of utoimmune is multisystem granulomatous disorder as cerebral syphilitic gumma and the definitive diagnosis still requires biopsy.

In clinical practice, cerebral syphilitic gumma remains a great imitator. A few cases reported diagnostic errors before surgery. In 2024, Mu et al. (2024) reported one case of cerebral syphilitic gumma misdiagnosed as brain abscess.

Advanced neuroimaging techniques such as MR spectroscopy, CEST, MR perfusion-weighted imaging, DCE-MRI and multiple-b values DWI provide new strategy for the diagnosis and differential diagnosis of brain lesions. While conventional MRI offers morphological characteristics, advanced neuroimaging techniques can reflect diffusion, perfusion and metabolic information and molecular features.

MR Spectroscopy Imaging (MRS) provides the change in metabolism and biochemistry in brain or tumor tissue by analyzing relative concentrations of metabolites NAA decrease corresponds to neuronal death or injury or the replacement of healthy neurons by other (e.g., tumor) cells. Cho increases whenever there is cellular proliferation, inflammation or demyelination. Lactate is not routinely detectable in healthy brain, and the probability to observe lactate increases with the grade of the tumor, as the results of anaerobic glycolysis. a Cho/NAA ratio greater than 1 is considered to be positive for neoplasm (Callot et al., 2008). CEST is a molecular MRI technique that can indirectly reflect endogenous cellular protein information (Zhou et al., 2019). The high cellularity, and over-expression of proteins in tumor tissue lead to CEST hyperintensity (Schon et al., 2020).

The MR spectroscopic findings in our case showed a mild increase in Cho as well as slight decreased Cr and NAA (Cho/Cr = 1.31; Cho/NAA = 1.72), which showed variation from the observation in a previously published case (Cui et al., 2020). The APTw showed high signal which suggested proteins over-expressed in the lesion.

DCE MRI primarily provides information on cerebral physiology at the capillary level (microvasculature). The majority of dural metastases show low perfusion results (Zimny and Sasiadek, 2011). Cerebral hemodynamic parameter suggested hypoperfusion through analysis. The above functional MR results may contribute to the exclusion of common intracranial neoplasia.

IVIM and SEM is an extension of the DWI that allows the simultaneous acquisition of both microcirculatory and diffusivity information (Hu et al., 2015). SEM is an advanced diffusion model that considers the composition of continuous distribution of ADC in each part. The high α and DDC values of the nodule indicate the high tissue heterogeneity and low level of diffusion restriction (Zheng et al., 2023). IVIM model separates microvascular fast diffusion from gaussian diffusion. The high level of D* and low level of f in the nodule reflects the microvascular obliteration, which is one of the key features of syphilis pathology.

Some cases (Noel et al., 2011; Mejdoubi et al., 2020) have been reported in the literature, which developed in the temporal lobe as in our case without abnormal auditory perceptions. In 2020, Xia et al. (2020) reported a patient with hypoacusis, while the lesion was located in cerebellopontine angle (CPA). The possible explanation for hearing loss in our case was otosyphilis rather than mass effect, which may cause by the chronic inflammatory processes of syphilis involving the VIII nerve or temporal bone (Lafond and Lukehart, 2006).

For patients with neurosyphilis and an undefined intracranial mass without trends of increased intracranial pressure, brain herniation, or progressive symptoms, to avoid unnecessary surgery, high-dose penicillin treatment can be attempted (Chow, 2021). And subsequent treatment plan can be revised based on therapeutic effectiveness by assessing dynamically observation of intracranial imaging changes (Keil et al., 2017).

## Conclusion

In summary, cerebral syphilitic gumma is exceedingly rare, presenting with diverse clinical manifestations, and lacks specific findings on routine imaging. Combined with unclear medical history and inadequate recognition of this condition among clinicians, misdiagnosis or missed diagnosis is common. Advanced neuro-MRI serves as a significant complement to conventional MRI imaging. It aids in avoiding unnecessary surgical treatment and offers new perspectives in distinguishing cerebral syphilitic gumma from other intracranial mass lesions.

## Data availability statement

The original contributions presented in the study are included in the article/Supplementary material, further inquiries can be directed to the corresponding author.

## Ethics statement

Written informed consent was obtained from the individual(s) for the publication of any potentially identifiable images or data included in this article.

## Author contributions

XS: Writing−original draft. ZZ: Writing−review & editing. XL: Writing−review and editing. WZ: Writing−review and editing. XZ: Writing−review and editing, Funding acquisition, Data curation. BZ: Writing−review and editing, Project administration, Data curation.
