# Supplementary material for: Contribution of advanced neuroimaging in diagnosis of cerebral syphilitic gumma: a case report
Source: Front Neurosci. 2024 Aug 14;18:1442176. doi: 10.3389/fnins.2024.1442176 (PMC11349654; doi:10.3389/fnins.2024.1442176)
Supplement: Supplementary file 1 [file Data_Sheet_1.docx]

Contribution of Advanced Neuroimaging in diagnosis of Cerebral Syphilitic Gumma: A Case Report

**Xinyi Shen1,2,3†, Zhengyang Zhu2,3†, Xin Li1,2,3, Wen Zhang1,2,3, Xin Zhang1,2,3*, Bing Zhang1,2,3,4,5**

^1^ Department of Radiology, Nanjing Drum Tower Hospital, Affiliated Hospital of Medical School, Nanjing University, Nanjing, China

^2^ Institute of Medical Imaging and Artificial Intelligence, Nanjing University, Nanjing, China

^3^ Medical Imaging Center, Affiliated Drum Tower Hospital, Medical School of Nanjing University, Nanjing, China

^4^ Jiangsu Key Laboratory of Molecular Medicine, Nanjing, China

^5^ Institute of Brain Science, Nanjing University, Nanjing, China

†These authors contributed equally to this work and share first authorship

*** Correspondence:** Xin Zhang**:** [neuro_zx@163.com](mailto:neuro_zx@163.com)

# Supplementary Data

All the preoperative MRI date were acquired using a 3.0T MRI scanner (uMR790, United Imaging Healthcare, Shanghai, China) with a 32-channel phased-array head coil. Conventional MRI examinations included 3D T1WI pre- and post- the injection of gadolinium-based contrast agent (repetition time [TR]/ echo time [TE]=7.9/3.1 milliseconds; inversion time [TI]=810 milliseconds; flip angle [FA]=10◦; matrix=256 × 256; field of view [FOV]=256 × 232 mm^2^; slice thickness=1 mm), 3D T2WI (TR/TE=2200/606.36 milliseconds; TI=1519 milliseconds; FA: from 19° to 150°; matrix=256 × 256; FOV=256 × 232 mm^2^; slice thickness=1 mm) and 3D FLAIR (TR/TE=4800/428.04 milliseconds; TI=1519 milliseconds; FA: from 21° to 150°; matrix=240 × 240; FOV=256 × 232 mm^2^; slice thickness=1mm).

Multi-b values DWI images were obtained using a single-shot spin-echo echo planar imaging sequence (SS SE-EPI) with 12 b-values (0, 20, 50, 100, 200, 400, 700, 1000, 1500, 2000, 2500 and 3000 s/mm^2^) in three directions. The detailed multi-b values DWI protocol was as follows: TR/TE= 3598/108.1 milliseconds; FA=90◦; matrix=144 × 144; FOV=230 × 230 mm^2^; slice thickness=5 mm. Intravoxel incoherent motion (IVIM) and stretched exponential model (SEM) metrics were calculated from multi-b values DWI images

Axial DCE-MRI acquisition was performed using dynamic scan of a T1-gradient echo sequence and setting the following parameters: TR/TE=3.47/1.9 milliseconds; FA=13°; matrix=160 × 160; FOV=240 × 220 mm^2^; slice thickness=5 mm. Pre-contrast images with multiple FA 5, 10 and 15° were acquired for the T1 maps. Then the contrast agent (Gadovist, 1 mmol/mL, Bayer Healthcare, Berlin, Germany) was administered (0.1mmol/kg of bodyweight) through the antecubital vein via a power injector at a rate of 2 mL/s. A series of 1800 images at 90 dynamic phases for 20 axial sections were obtained with a temporal resolution of 4 seconds for each dynamic phase.

DCE, IVIM and SEM metrics were calculated using United imaging software workstation. For the IVIM model, DWI signal intensities at multiple b-values were fitted with the following equation:

$$\frac{S_{b}}{S_{0}}=F*\exp\left( -b*D^{*} \right)+\left( 1-f \right)*exp(-b*D)$$

Where S(b) is the signal intensity at a given b-value; D and D* are the diffusion coefficient and pseudo-diffusion coefficient which is related to the tissue diffusion and microvascular perfusion; F is the IVIM-based perfusion fraction.

For the SEM model, DWI signal intensities at multiple b-values were fitted with the following equation:

$$\frac{S_{b}}{S_{0}}=\exp\{-\left( b*{DDC}^{\alpha} \right)\}$$

Where S(b) is the signal intensity at a given b-value; DDC is the mean intravoxel diffusion rate and α is related to the intravoxel water heterogeneity, varying between 0 and 1.

The following DCE parameters were analyzed based on the Tofts model: Ktrans, Kep, Ve and iAUC. Ktrans refers to the volume transfer constant, signifying the flow of gadolinium from the blood plasma into the extravascular extracellular space (EES); Kep denotes the time constant of gadolinium reflux from the EES back into the vascular system; Ve represents the EES volume per unit tissue volume and iAUC illustrates to the initial area under the time-concentration curve for the first 60 seconds.
